# Late auditory event-related evoked potential (P300) in Down's syndrome patients

**DOI:** 10.1590/S1808-86942010000200010

**Published:** 2015-10-19

**Authors:** Carla Patrícia Hernandez Alves Ribeiro César, Heloisa Helena Caovilla, Mário Sérgio Lei Munhoz, Maurício Malavasi Ganança

**Affiliations:** Doctorate in the science of communication disorders, Sao Paulo Federal University, Paulista Medical School. Professor and coordinator of the speech therapy course at the Sao Paulo Methodist University; Speech therapist; doctoral degree; associate professor of the otology and otoneurology discipline at the Sao Paulo Federal University, Paulista Medical School; Physician, otorhinolaryngologist, doctoral degree. Associate professor of the otology and otoneurology discipline at the Sao Paulo Federal University, Paulista Medical School; Physician, otorhinolaryngologist, doctoral degree. Full professor of the Sao Paulo Federal University, Paulista Medical School. Professor of the master's degree program on vestibular rehabilitation and social inclusion of the Sao Paulo Bandeirante University. Sao Paulo Methodist University

**Keywords:** down syndrome, p300, event-related potentials

## Abstract

Down syndrome is caused by a trisomy of chromosome 21 and is associated with central auditory processing deficit, learning disability and, probably, early-onset Alzheimer's disease.

**Aim:**

to evaluate the latencies and amplitudes of evoked late auditory potential related to P300 events and their changes in young adults with Down's syndrome.

**Materials and Method:**

Prospective case study. P300 test latency and amplitudes were evaluated in 17 individuals with Down's syndrome and 34 healthy individuals.

**Results:**

The P300 latency (N1, P2, N2 and P3) was longer and the N2-P3 amplitude was lower in individuals with Down syndrome when compared to those in the control group.

**Conclusion:**

In young adults with Down syndrome, N1, P2, N2 and P3 latencies of late auditory evoked potential related to P300 events were prolonged, and N2 - P3 amplitudes were significantly reduced, suggesting integration impairment between the auditory association area and cortical and subcortical areas of the central nervous system.

## INTRODUCTION

Langdon Down described Down's syndrome, or the chromosome 21 trisomy, over a century ago; it is considered the most common genetic cause of mental retardation.^1^ The typical chromosomal changes in this syndrome comprise a homogeneous group^2^ that makes it easy to understand the findings. Diffuse injury or dysfunction in certain central areas,^3^ failure in habituation mechanisms,^4^ poor central inhibition of afferent stimuli,^5^ or cognition disorders^6^ may give rise to decreased brain electrical activity in Down's syndrome.

The long latency event-related evoked potential (P300) is associated with cognition;^7^ it is generated when a random infrequent stimulus is detected among a series of frequent stimuli.^7^, ^8^, ^9^ The parameters assessed are latency and amplitude. Latency varies according to an individual's difficulty in discriminating a rare tones among frequent stimuli; amplitude varies according to the improbability of the stimulus.^8^ Waves N2 and P3 are the most important because they represent physiological phenomena associated with memory and learning mental events.^10^ Wave N2 is associated with decision-making or discrimination of stimuli;^11^ absent N2 and P3 waves suggest more severe involvement, such as pre-senile or senile dementia.^12^

Increased P3 latency and decreased P3 amplitude^4^, ^5^^,^^13^^,^^14^, ^15^, ^16^, ^17^ have been identified in Down's syndrome. Increased latency appears to be associated with sensory and cognitive deficiencies in information processing, in particular by delaying the categorization of auditory information, and may be due to differences in the brain's ability to organize and respond in Down's syndrome.^14^

Increased P3 latency and decreased P3 amplitude, seen in healthy ageing individual,^18^, ^19^ have been found at an earlier age - about 20 years earlier^15^^,^^20^ - in patients with Down's syndrome. A line of theory considers the possibility of an Alzheimer's type pre-senile dementia in subjects with Down's syndrome, underlining the importance of P300 to investigate the triad: ageing/Down's syndrome/Alzheimer's disease.^13^

The lack of Brazilian papers on the use of P300 in Down's syndrome motivated this study.

The purpose of this study was to assess late event-related auditory evoked potentials (P300) in young adults with Down's syndrome.

## MATERIALS AND METHODS

The Institutional Review Board of the institution in which the investigation was carried out approved this study (number 181/96).

We undertook a prospective study comprising subjects aged from 18 to 39 years with a diagnosis of Down's syndrome. These patients were referred from a specialized institution on this condition; their parents or caretakers gave their free permission for each patient to participate. There were 17 subjects (10 male and seven female). Parents or caretakers of the subjects were informed of the study procedures and signed a free informed consent form for P300 to be done on the patients.

The control group consisted of healthy subjects with no neurological or auditory complaints and no history of (or suspected history of) first degree family members with dementia. The control group comprised 34 subjects (20 male and 14 female) aged from 18 to 39 years. This group had twice the number of patients in the Down's syndrome group, to increase test reliability.

Subjects in both groups were asked to refrain from stressful physical and mental activities before undergoing the auditory P300 testing. Those that did not follow this instruction or that slept during the procedure were excluded from the sample.

The Nicolet Compact Auditory (Nicolet Biomedical Instruments) ran the P300 program with the following parameters: pure tone stimulus, frequent tone (EF) at 750 Hz, and rare tone (ER) at 2,000 Hz; binaural presentation mode; 70 dBHL intensity with 700 ms intervals between tones; and a 4/1 proportion for randomly presenting EF/ER (odd ball paradigm). Electrodes were placed on Cz (A1 and A2) and Pz (A1 and A2). TDH-39 earphones were used. About 300 stimuli were presented.

Testing took place in an acoustically isolated and semi-darkened silent room; patients were comfortably seated on a reclining seat with head support. The skin and scalp were cleaned with an abrasive paste (OMNI), after which electrolytic gel was placed between the skin and electrodes to increase conductivity. Electrodes were fixed with microporeT plaster on the frontal (Fpz), vertical (Cz) and parietal (Pz) regions, and left ear (A1) and right ear (A2) lobules, according to the 10–20 international standard.21 The electrodes were connected to a preamplifier, in turn connected to the subject's clothes. Electrode polarity was positive in Cz and Pz, and negative in A1 and A2 (interlinked with a jumper). The electrical impedance was less than 5 KOhms in each derivation, and less than 3 KOhms between two different derivations throughout the procedure.

Electroencephalogram waves were amplified 10,000 times; the bandwidth ranged from 1 to 30 Hz; the sensitivity was about 50mv. Artifacts were rejected by an internal circuit in the device that excluded all recordings with potentials over 45mv from the mediation process. Testing was interrupted if artifacts surpassed 20% of the presented stimuli, and the patient was asked to avoid muscle tension, and eye and body movements; after a 5-minute rest period, the test was restarted.

Study subjects were asked to remain alert and relaxed for testing. They had to raise their hands or a flag gently only for rare tones to demonstrate tone discrimination and attentiveness. At the beginning of the test some of the subjects with Down's syndrome required new instructions, as they raised their hands or the flag and did not bring it down afterwards; the test was restarted only after it was fully understood.

Each procedure lasted about thirty minutes: 15 minutes were required for preparing and placing the electrodes, data entry, and checking the impedance; 3 minutes were needed for the pre-test phase, during which the examiner identified rare tones with each subject until he or she could perform it alone; the test itself took from seven to 10 minutes.

Waves N1, P2, N2 and P3 had, respectively, negative-positive-negative-positive polarities, and were recorded and replicated when frequent and rare tones were presented. N1 and P2 were marked on the frequent tones, and N1, N2 and P3 were marked on rare tones. Latency was measured at the maximum deflection point in each wave, and amplitude was measured between the maximum N1 and P2, and N2 and P3 deflection points. Peaks with the highest deflection or waves that were best duplicated were measured in double, triple or multiple peak waves.^22^

P300 component latency and amplitude values were tabulated for the control group and the Down syndrome group. The mean latencies and amplitudes in each group were compared and related with gender. The Mann-Whitney test, used for comparing independent samples, was applied for the statistical analysis. The significance level was 5%. Significant data were marked with an asterisk.

## RESULTS

There were 51 subjects aged from 18 to 39 years; 30 were male and 21 were female. All were able to perform the test without difficulty.

Table 1 shows N1, P2, N2 and P3 latency values and N1-P2 and N2-P3 amplitude values for 17 subjects with Down's syndrome. Table 2 shows N1, P2, N2 and P3 latency values and N1-P2 and N2-P3 amplitude values of 34 controls.

**Table 1:** Values of N1, P2, N2 and P3 latencies and N1-P2 and N2-P3 amplitudes in 17 subjects with Down's syndrome.

Patient	Age	N_1_ Latency	P_2_ Latency	N_2_ Latency	P_3_ Latency	N_1_-P_2_ Amplitude	N_2_-P_3_ Amplitude
S_1_	19	112,0	198,4	457,6	540,8	14,2	6,0
S_2_	20	102,4	172,8	272,0	326,4	18,0	3,9
S_3_	21	108,8	179,2	275,2	329,6	4,1	6,2
S_4_	21	102,4	176,0	236,8	307,2	6,6	5,8
S_5_	23	105,6	153,6	281,6	374,4	5,8	4,3
S_6_	24	112,0	192,0	240,0	355,2	8,4	11,9
S_7_	25	89,6	153,6	294,4	364,8	8,4	5,3
S_8_	25	112,0	198,4	272,0	320,0	3,3	3,7
S_9_	25	108,8	150,4	243,2	361,6	5,8	3,5
S_10_	25	86,4	192,0	307,2	345,6	12,1	2,7
S_11_	26	99,2	179,2	256,0	313,6	10,1	9,0
S_12_	27	102,4	176,0	291,2	342,4	10,7	2,9
S_13_	27	99,2	192,0	–	–	10,7	–
S_14_	27	105,6	188,8	272,0	348,8	11,1	4,7
S_15_	27	108,8	150,4	252,8	310,4	8,4	5,8
S_16_	35	115,2	185,6	259,2	297,6	8,2	1,9
S_17_	38	105,6	176,0	396,8	476,8	4,3	3,3
Mean	25,6	104,5	177,3	288,0	357,2	8,9	5,1

KEY: S_number_ = Subject_number_

**Table 2:** Values of N1, P2, N2 and P3 latencies and N1-P2 and N2-P3 amplitudes in 34 healthy subjects in the control group.

Patient	Age	N_1_ Latency	P_2_ Latency	N_2_ Latency	P_3_ Latency	N_1_-P_2_ Amplitude	N_2_-P_3_ Amplitude
S_1_	18	86,4	118,4	144,0	320,0	2,7	12,9
S_2_	18	99,2	163,2	201,6	243,2	9,0	1,4
S_3_	21	92,8	160,0	195,2	278,4	3,1	9,0
S_4_	22	131,2	156,8	211,2	291,2	3,5	16,4
S_5_	22	89,6	172,8	233,6	265,6	11,1	0,8
S_6_	22	102,4	192,0	300,8	352,0	9,8	7,2
S_7_	23	96,0	134,4	182,4	243,2	8,0	10,1
S_8_	23	89,6	166,4	214,4	300,8	6,8	14,8
S_9_	23	96,0	211,2	272,0	300,8	16,0	1,7
S_10_	23	108,8	166,4	217,6	336,0	7,6	8,6
S_11_	23	99,2	172,8	198,4	300,8	9,7	3,7
S_12_	24	92,8	140,8	179,2	224,0	5,3	12,3
S_13_	24	99,2	144,0	198,4	345,6	7,0	9,8
S_14_	24	96,0	163,2	201,6	265,6	10,0	9,0
S_15_	24	96,0	115,2	137,6	262,4	1,4	16,0
S_16_	25	96,0	140,8	185,6	252,8	3,5	20,1
S_17_	25	102,4	160,0	188,8	236,8	6,0	7,0
S_18_	25	89,6	153,6	224,0	284,8	10,1	14,8
S_19_	26	96,0	147,2	195,2	220,8	15,0	4,5
S_20_	26	96,0	176,0	230,4	291,2	11,5	5,7
S_21_	26	99,2	160,0	204,8	243,2	4,3	8,8
S_22_	26	83,2	156,8	214,4	304,0	10,0	10,9
S_23_	26	96,0	179,2	233,6	307,2	9,2	10,1
S_24_	26	102,4	150,4	195,2	297,6	8,8	7,8
S_25_	26	89,6	115,2	163,2	272,0	2,3	10,9
S_26_	27	115,2	160,0	195,2	304,0	7,8	20,9
S_27_	27	108,8	163,2	201,6	291,2	6,4	3,1
S_28_	27	105,6	150,4	211,2	300,8	8,0	10,5
S_29_	29	108,8	140,8	172,8	236,8	4,1	10,1
S_30_	29	99,2	160,0	217,6	294,4	6,8	10,5
S_31_	31	115,2	137,6	166,4	268,8	1,9	13,7
S_32_	36	99,2	204,8	259,2	307,2	11,5	6,4
S_33_	36	99,2	198,4	297,6	358,4	12,1	9,8
S_34_	39	96,0	150,4	195,2	307,2	10,0	0,6
Mean	25,6	99,2	158,3	207,1	285,6	7,7	9,4

KEY: S_number_ = Subject_number_

Tables 3 and 4 show the influence of gender on N1, P2, N2 and P3 latency values and N1-P2 and N2-P3 amplitude values of 17 with Down's syndrome and of controls. The Mann-Whitney test, at a 5% significance level, revealed no statistically significant gender differences.

**Table 3:** Mean values of age, N1, P2, N2 and P3 latencies, and N1-P2 and N2-P3 amplitudes of 17 subjects with Down's syndrome according to gender.

Parameters	Gender Male Mean Values	Gender Female Mean Values	p - value
Age	26,8	23,9	0,2768
N_1_ latency	103,0	106,5	0,5540
P_2_ latency	180,2	173,3	0,3019
N_2_ latency	303,6	267,9	0,5593
P_3_ latency	364,1	348,3	0,3683
N_1_ - P_2_ amplitude	8,6	9,1	0,9609
N_2_ - P_3_ amplitude	4,9	5,3	0,7911

Mann-Whitney test

**Table 4:** Mean values of aged, N1, P2, N2 and P3 latencies and N1-P2 and N2-P3 amplitudes of 34 controls according to gender.

Parameters	Gender Male Mean Values	Gender Female Mean Values	p - value
Age	26,6	24,2	0,1207
N_1_ latency	99,2	99,2	0,7899
P_2_ latency	157,9	158,9	0,9580
N_2_ latency	201,9	214,4	0,1456
P_3_ latency	285,3	285,9	0,8885
N_1_ - P_2_ amplitude	7,0	8,6	0,1414
N_2_ - P_3_ amplitude	9,8	8,9	0,6743

Mann-Whitney test

Table 5 shows a comparison of N1, P2, N2 and P3 mean latencies between both groups. The Down's syndrome group showed a statistically significant increase in N1, P2, N2 and P3 latencies compared to controls.

**Table 5:** Comparison of mean N1, P2, N2 and P3 latencies of 17 subjects with Down's syndrome and 34 controls.

Latency	Down's syndrome Mean Values	Control Group Mean Values	p - value
N1	104,5	99,2	0,0088*
P2	177,3	158,3	0,0032*
N2	288,0	207,0	0,0000*
P3	357,2	285,5	0,0000*

Mann-Whitney test

Table 6 shows a comparison of mean N1-P2 and N2-P3 amplitudes in 17 subjects with Down's syndrome and 34 controls. A statistically significant difference was found in N2-P3 amplitudes, which were lower in the Down's syndrome group compared to controls.

**Table 6:** Comparison of mean N1-P2 and N2-P3 amplitudes of 17 subjects with Down's syndrome and 34 controls.

Amplitudes	Down's syndrome Mean Values	Control Group Mean Values	p - value
N1-P2	8,8	7,7	0,3224
N2-P3	5,0	9,4	0,0023*

Mann-Whitney test

## DISCUSSION

In this study, P300 was easy to apply and interpret in subjects with Down's syndrome. All study and control subjects avoided physical activities and stressful mental work before undertaking the auditory P300 test; we were careful to exclude from the sample the subjects that did not follow the instructions, or that were fatigued, inattentive, or that slept during the procedure. The parameters that were assessed were N1, P2, N2 and P3 latencies and N1-P2 and N2-P3 amplitudes. Of 17 subjects with Down's syndrome, 12 (70.6%) showed one or more abnormalities in N2 and P3 latencies and/or N1-P2 and N2-P3 amplitudes. We found delayed N2 latency in 29.4% of cases, delayed N2 and P3 latencies in 23.5%, delayed P3 latency in 5.9%, delayed N2 latency and increased N1-P2 amplitude in 5.9%, and absent N2 and P3 components in 5.9%, compared with the results of healthy subjects^23^ using the same procedure of raising one's hand upon hearing a rare tone.

We found that N1, P2, N2 and P3 latencies were higher in the Down's syndrome group, which concurred with other studies.^5^, ^6^^,^^16^ The difference of the mean P3 latency between subjects with Down's syndrome and controls was 71 ms, suggesting that the estimated mean age of Down's syndrome subjects would be 96 years,^22^ which contrasts with their mean reported chronological age (25 years). The literature we reviewed suggested that the P300 test would demonstrate the effects of human ageing,^18^ observed in a slowing of the stimulus assessment process and decreased selective attention ability;^24^ P3 latency increases by about one millisecond a year throughout life.^22^ As the P3 latency is elevated in healthy elderly subjects^19^ and in Down's syndrome subjects,^5^^,^^13^ we assumed that the early ageing hypothesis^17^ that is reported in the literature could be applied to our study group. However, the elevation in P3 latency we found might also be related only with sensory and cognitive information processing deficiencies, which would delay categorization of auditory information, and which may have been attributed to differences in the ability of the brain of Down's syndrome subjects to organize information and respond.^14^

N1-P2 amplitudes were similar in subjects with Down's syndrome and controls, while N2-P3 amplitude was lower in the Down's syndrome group. These results concur with other findings in the literature.^16^, ^17^ As amplitude decreases with ageing in healthy subjects,^18^, ^19^ our findings in some subjects with Down's syndrome led us to hypothesize the possibility of early ageing, compared to controls, and point to the neurological and cognitive losses in our study sample. Decreased N2-P3 amplitude might suggest that few fibers are conducting the stimuli, indicating that there might be loss of auditory attentiveness^25^ and poor cognition.^7^

Altered P300 latencies and amplitudes may be justified in different theoretical perspectives; there might be failure in cortical habituatioin,^4^, ^5^, ^6^ a decreased number of nervous fibers for conducting auditory stimuli,^25^ the cognitive disorder itself,^7^ or the possibility of Alzheimer's disease in individuals with Down's syndrome.^16^

Identifying altered P300 latencies and amplitudes in subjects with Down's syndrome demonstrates the importance of this procedure, suggesting that surveys to monitor the progression of these cases could be undertaken to clarify the behavior of mentally retarded individuals without Down's syndrome, of individuals with Alzheimer's disease, of individuals with neuroendocrine disorders, and of healthy individuals.

## CONCLUSION

In young adults with Down's syndrome N1, P2, N2 and P3 latencies were elevated and N2-P3 amplitudes were decreased in the event-related long-latency auditory evoked potential (P300), suggesting loss of integration in the auditory association area with cortical and subcortical areas of the central nervous system.
